# Analytical considerations in the determination of uranium isotope ratios in solid uranium materials using laser ablation multi-collector ICP-MS

**DOI:** 10.1016/j.acax.2019.100018

**Published:** 2019-04-30

**Authors:** Michael Krachler, Zsolt Varga, Adrian Nicholl, Klaus Mayer

**Affiliations:** European Commission, Joint Research Centre (JRC), Karlsruhe, Germany

**Keywords:** MC-ICP-MS, Laser ablation, Uranium isotopes, Inhomogeneity, Nuclear forensics

## Abstract

Validated analytical measurement protocols for the fast and accurate determination of the uranium (U) isotopic composition (^234^U, ^235^U, ^236^U, ^238^U) of solid nuclear materials were developed employing ns-laser ablation (LA) coupled to multi-collector ICP-MS. The accuracy of the analytical procedure was assured by frequent (*n* = 65) analysis of a pressed pellet of certified isotopic reference material CRM U-030 (∼3 wt% ^235^U). The expanded uncertainty (*k* = 2) for the *n*(^235^U)/*n*(^238^U) ratio was as low as 0.05%, rising to 0.62% and 1.09% for *n*(^234^U)/*n*(^238^U) and *n*(^236^U)/*n*(^238^U) ratios, respectively. LA-MC-ICP-MS measurements of a pressed pellet of certified isotopic reference material CRM U-020 (∼2 wt% ^235^U) before analysis of each sample allowed calculation of the ion counter gains and mass bias correction. Both individual spot analysis and line scan analysis were used to measure *n*(^234^U)/*n*(^238^U), *n*(^235^U)/*n*(^238^U), and *n*(^236^U)/*n*(^238^U) ratios in two low-enriched UO_2_ pellets from the fourth Collaborative Materials Exercise (CMX-4), four seized low-enriched UO_2_ pellets intercepted from illicit trafficking and one metal sample consisting of depleted U. LA-MC-ICP-MS results of all investigated samples matched well with U isotope ratios obtained by thermal ionisation mass spectrometry (TIMS). This independent confirmation of the LA-MC-ICP-MS results by TIMS underpinned the high quality of generated analytical data. Acquisition of several thousand data points within a couple of minutes during line scan analysis yielded detailed information on the spatial distribution of the U isotopic composition of selected UO_2_ pellets, revealing straightforwardly their (in˗)homogeneity on the μm-scale. Calculating skewness and half width of the frequency distributions of the *n*(^235^U)/*n*(^238^U) amount ratio allowed the quantitative assessment of the (in-)homogeneity of the investigated samples. This information allows drawing conclusions on the starting materials used for the production of the pellets. From a nuclear forensics perspective, LA-MC-ICP-MS provides quick, accurate results on the spatial distribution of major and minor U isotopes while preserving the sample i.e. piece of evidence, essentially intact.

## Highlights

• Accurate analysis of major and minor abundant U isotopes in various solid U materials.
• LA-MC-ICP-MS results of homogenous samples confirmed by independent TIMS measurements.
• Spatially resolved minor U isotope ratios.
• Assessment of isotopic inhomogeneity of U materials using descriptive statistics.

## Graphical abstract

**Figure:**
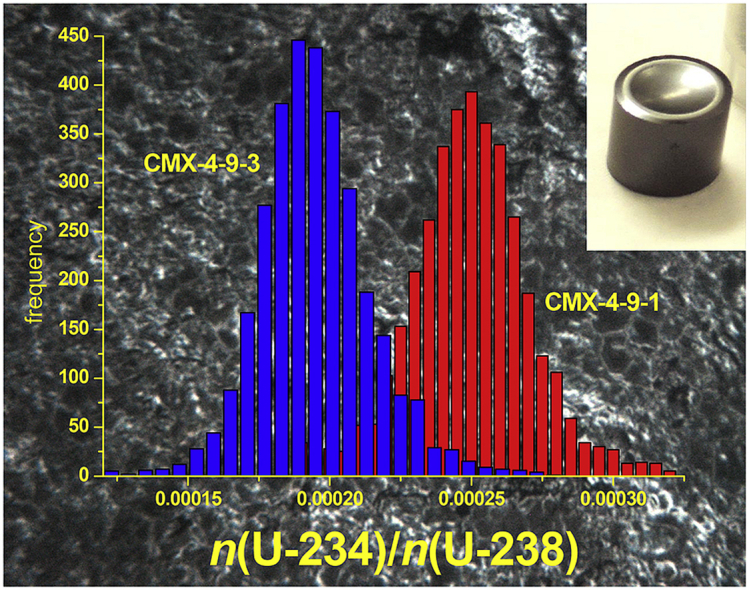

## 1 Introduction

In order to support investigations of unlawful or criminal incidents involving nuclear and other radioactive material, information on the history and possible origin of the illicit material needs to be obtained. To this end, trustworthy analytical data, characterising seized specimens, are required [1,2]. This branch of science, referred to as nuclear forensics, aiming at identifying the origin of nuclear material found out of regulatory control, is increasingly gaining importance as an essential nuclear security tool [1,2].

In this context, accurate and precise U isotopic data of a seized material, delivered in a timely manner, is a key parameter. To foster the capability of nuclear forensics laboratories to provide such high quality data, the Nuclear Forensics International Technical Working Group (ITWG) organises regularly Collaborative Materials Exercises (CMXs) to identify and share best analytical approaches in this scientific area among the participants [3,4].

Several laboratories dealing with U isotopic analysis of nuclear materials apply destructive analysis, i.e. a fraction of the solid sample is dissolved in strong acids, followed by chemical separation of U from the sample matrix constituents [5,6]. The actual measurement step is conducted using either thermal ionisation mass spectrometry (TIMS), high-resolution ICP-OES [7,8], or any kind of ICP-MS instrumentation [5,6]. This analytical approach, especially in combination with TIMS, is well established and offers very accurate and precise results. On the other hand, it requires labour intense chemical treatment which “destroys” irretrievably the sample. Moreover, such an analytical approach, results in an average U isotopic composition of the bulk material. The same applies for non-destructive high resolution gamma spectrometry [9]. Information on potential sample inhomogeneity, however, is not measurable by these methods.

To overcome the above mentioned drawbacks, laser ablation (LA), coupled to either single or multi-collector ICP-MS, is increasingly employed for U isotopic analysis of nuclear samples [[10], [11], [12], [13], [14], [15], [16], [17], [18], [19], [20]]. As LA does not require any sophisticated sample preparation, measurement results can be obtained in a timely manner. The sample remains virtually intact because LA requires only a few ng of solid material for subsequent mass spectrometric analysis [20]. Additionally, spatial U isotopic information gained through LA line scan analysis provides valuable hints on the homogeneity of the material in question [21].

The variety of diverse U isotopic compositions of solid nuclear materials can be also assessed with secondary ion mass spectrometry (SIMS). This was demonstrated during the fourth Collaborative Materials Exercise (CMX-4) where U particles were collected from a pellet surface on a cotton swipe by wiping the solid sample [22]. These particles were subsequently analysed individually by SIMS. As such, micro beam analysis of individual particles delivers accurate and precise information on major (^235^U and ^238^U) and minor (^234^U and ^236^U) abundant U isotopes [22]. It should be noted, however, that such SIMS analysis measures particles that are generally removed from the sample surface, while LA-(MC-)ICP-MS is generating spatially resolved U isotopic data directly from the solid sample. Therefore, LA-MC-ICP-MS and SIMS data sets on the same sample might not match perfectly. Instead, the information gained from both analytical approaches will complement each other.

In continuation of our work on the exploration of the potential of LA-ICP-MS [10,11] and LA-MC-ICP-MS [20,21] for the U isotopic analysis of solid nuclear materials, this study also included the analysis of the minor U isotopes, i.e. ^234^U and ^236^U. LA-MC-ICP-MS experiments aimed at the simultaneous, accurate measurement of ^234^U, ^235^U, ^236^U, and ^238^U in selected nuclear specimens comprising depleted as well as low-enriched U. The developed measurement protocols were applied to two low-enriched UO_2_ pellets from CMX-4 and five seized nuclear materials, i.e. various UO_2_ pellets and U metal. Experimental results allowed a direct comparison of spatial LA-MC-ICP-MS analysis to TIMS data, the latter signifying the U isotopic composition of the bulk material. Based on these overall results, this investigation shed light on the accuracy and precision of U isotopic analysis achievable with various analytical approaches and discusses the crucial issue of representative sampling. The degree of isotopic inhomogeneity of the investigated samples was assessed quantitatively using descriptive statistical tools such as skewness and half width of the frequency distributions of the *n*(^235^U)/*n*(^238^U) amount ratio.

## 2 Experimental

### 2.1 Instrumentation

Uranium isotopic measurements were carried out employing a double-focussing multi-collector inductively coupled plasma mass spectrometer (MC-ICP-MS, NuPlasma™, NU Instruments, Wrexham, Wales, U. K.). The instrument was operated in low mass resolution mode (*R* = 300) with the major U isotopes (^235^U and ^238^U) measured on Faraday detectors, while ion counters were used for the measurement of the minor abundant isotopes ^234^U and ^236^U. Before actual sample measurements, ion counters were checked for accurate dead time correction and appropriate plateau voltage settings. The application of a retardation filter for the ion counter measuring ^236^U effectively helped to minimise the impact of potential peak tailing of the large ^235^U and ^238^U signals into the ^236^U peak [20,23].

Minute amounts of solid materials were sampled from their surfaces using a ns-laser ablation unit (ESI Lasers, Bozeman, MT, U.S.A.) operated at a wavelength of 213 nm. The ns-laser ablation unit was equipped with a two-volume cell (a so-called TV2 cell) largely helping to eliminate potential cross-contamination of other samples within the LA chamber. The generated particle plume was transported from the LA cell to the plasma of the MC-ICP-MS using a laminar flow of Ar gas. Solutions were aspirated into the plasma of the MC-ICP-MS through a desolvating nebuliser (Aridus II, Cetac Technologies Inc. Omaha, NE, USA). Both sample introduction systems (for liquid and solid samples) were coupled to each other via a Y-connection. Further details on the optimised instrumental settings as well as on the applied data acquisition parameters were reported elsewhere [20,21].

Powdered certified isotopic reference materials were pressed to pellets with an X-PRESS hydraulic laboratory press (Spex Industries, Metuchen, USA) for straightforward handling and to avoid cross-contamination in the LA cell [20]. No kind of binder was added to the original powders during the preparation of the pellets [20].

### 2.2 Standards

An aliquot of the multi-element solution IV (Inorganic Ventures, Christiansburg, USA) containing 50 ng g^−1^ U was employed for daily optimisation of the performance of the MC-ICP-MS.

The accuracy of U isotope measurements of solid materials was assured through regular analysis of pressed CRM U-030 (NBL, Argonne, IL, USA). Similarly, pressed CRM U-020 (NBL, Argonne, IL, USA) was used to correct the U isotope ratio measurements in the samples for mass bias and to calculate the gain of the employed ion counters.

### 2.3 Investigated samples

Two low-enriched UO_2_ pellets from the fourth Collaborative Materials Exercise (CMX-4) organised by the International Technical Working Group (ITWG) on Nuclear Forensics, thereafter named CMX-4-9-1 and CMX-4-9-3, were included in this study [3]. Additionally, four low-enriched UO_2_ pellets (intercepted from illicit trafficking) of different origin and diverse U isotopic composition as well as one seized metal sample consisting of depleted U were analysed.

### 2.4 Measurement procedures

The MC-ICP-MS was set up for the simultaneous determination of major and minor U isotopes. Argon gas flows of both LA and MC-ICP-MS were optimised simultaneously aspirating a U standard solution. Signals of ^236^U were corrected for hydride formation (^235^U^1^H) via monitoring *m*/*z* 238 and *m*/*z* 239 (^238^U^1^H). All LA-MC-ICP-MS measurements were carried out on “native” samples, i.e. without any pre-cleaning of the sample surfaces.

Prior to a measurement sequence, the MC-ICP-MS was tuned and optimised with aqueous standard solutions. For LA measurements, only solid samples were analysed with no solution being aspirated via the desolvating nebuliser. As exclusively solid samples were analysed throughout this study, solid, matrix-matched certified reference materials were employed correspondingly for mass bias correction, for calculation of the gain of the employed ion counters and for quality assurance, as described in detail below. Small (∼5 μm) spots were ablated with the laser from the surface of solid samples placed in the LA cell. Their corresponding U isotopic ratios were determined by either single spot or line scan analysis. During single spot analysis, circular spots with diameters of 5 μm–10 μm were ablated at a frequency of 4 Hz for a period of 5 s. The laser was operated with a fluence (laser power) varying between 2 and 13 J cm^−2^ to ensure the ^238^U ion current remains within the linear range of the detector-amplifier system of the MC-ICP-MS, i.e. the signal not to exceed 10 V on the Faraday detector.

For the seized metal specimen consisting of depleted U, the measurement parameters were different. Applying the same measurement conditions used for the analysis of the various UO_2_ pellets to the seized metal sample, yielded largely varying U ion currents between individual spots. To compensate for that “variability” and to obtain adequate measurement performance, a larger surface area (spots of 50 μm diameter) was sampled at lower frequency (1 Hz) to smooth the obtained U signal. Additionally, the ablation time was doubled to 10 s, while the fluence was kept low, at ∼2 J cm^−2^.

For line scan analysis spots with a diameter of 5 μm were continuously ablated from the sample surface for several minutes while the focussed laser beam was moved along a pre-defined line with a speed of 20 μm s^−1^ at 4 Hz across the surface of the solid materials. The fluence of the laser (2–15 J cm^−2^) was adjusted to generate a^238^U signal of 4–8 V.

Raw data from MC-ICP-MS measurements were evaluated using Microsoft Excel^®^. Additionally, the entire data set was checked for outliers yielding the results reported here. The statistical evaluation of the data was performed with OriginPro^®^ (OriginLab Corporation, Northampton, USA).

### 2.5 Laser ablation cell design

A key requirement for obtaining high spatial resolution of the acquired LA-MC-ICP-MS data during line scan analysis is a short response time of the applied analytical set-up. In this respect, the two-volume LA cell (TV2 cell) employed in this study is well suited because it is characterised by a quick washout time [20]. Conventional LA cells sample the entire cell volume, while the improved design of the TV2 cell minimises the volume that is probed during measurements to an absolute minimum. In other words, the particle cloud generated during LA is diluted only into a small volume leading to a fast response of the recorded ICP-MS signals. Thus, the use of the TV2 cell provides higher sensitivity (less dilution of the “sample” compared to conventional LA cells) and avoids cross-contamination with other samples present in the LA cell.

### 2.6 Mass bias correction

Raw data were corrected for instrumental mass bias by measuring the *n*(^235^U)/*n*(^238^U) ratio in CRM U-020 prior to each sample measurement and applying the exponential law. Compared to the ion counter gains (see below), the mass bias was quite stable and typically varied within a range of ±0.25% throughout a measurement day. In general, the utilised mass bias correction method worked well, no matter if pressed U_3_O_8_ powders or sintered UO_2_ pellets were used for this purpose [20,21].

### 2.7 Ion counter gains

Because of their low abundance in the investigated samples, the respective ICP-MS signals of ^234^U and ^236^U needed to be measured with ion counters. While uncertainty contributions from the plasma or laser are the most significant limitation to improved accuracy and precision of the major abundant isotopes [24], the accuracy of the minor U isotope ratio measurements also depends on the accuracy of the relative efficiency (gain) calibration of the ion counters. This assessment needs to be repeated regularly because ion counter gains might drift during a measurement sequence, e.g. the age and state of the dynode of each ion counter as well as warm up time and signal level may influence this drift. To account for that, certified U reference materials producing well measurable ion currents for ^234^U and ^236^U were used.

In our experiments, a pressed pellet of CRM U-020 (∼2 wt% ^235^U) was analysed prior to any unknown sample for calculation of the ion counter gains. This suggested frequent renormalisation of the system response to an isotopic standard [24] was important because the response of the two employed ion counters varied ∼4% (^234^U) and ∼9 %–17% (^236^U) over an entire measurement day.

### 2.8 Quantitative assessment of spatial isotopic homogeneity

The *n*(^235^U)/*n*(^238^U) amount ratio of all measured data obtained from line scan analysis of the investigated samples was used to calculate the skewness of the distribution. Additionally, the frequency distribution of the *n*(^235^U)/*n*(^238^U) ratio was fitted with a Gaussian function from which the half width was subsequently calculated. The half width was expressed as full width at half maximum (FWHM). The skewness and half width of the distribution of the *n*(^235^U)/*n*(^238^U) ratio calculated from line analysis of two certified UO_2_ reference materials employed in a previous study [21] served as a point of reference for the assessment isotopic homogeneity of the samples included in this study.

## 3 Results and discussion

### 3.1 Quality control

The accuracy of all U isotope ratios measured in unknown samples using LA-MC-ICP-MS was verified through repeated analysis (*n* = 65) of the certified U isotopic standard reference material CRM U-030 in each measurement sequence (Table 1). The experimental average of the *n*(^234^U)/*n*(^238^U), *n*(^235^U)/*n*(^238^U) and *n*(^236^U)/*n*(^238^U) amount ratios of CRM U-030 agreed well with the certified values within the stated analytical uncertainties. To avoid cross-contamination with other samples simultaneously present in the LA cell the originally powdered CRM was pressed into a 5-mm pellet for easy and straightforward handling of the radioactive material. As such, CRM U-020 and CRM U-030 could be placed in the LA cell together with several samples and both CRMs could be measured easily at regular intervals.

**Table 1:** Certified and measured (*n* = 65) U isotopic composition (amount ratio ± uncertainty, wt%, *k* = 2) of a pressed pellet of certified reference material CRM U-030 as determined regularly on individual 5 μm spots on each day during the entire measurement campaign using LA-MC-ICP-MS.

	*n*(^234^U)/*n*(^238^U)	*n*(^235^U)/*n*(^238^U)	*n*(^236^U)/*n*(^238^U)
Certified	0.0001960 ± 0.0000010	0.031430 ± 0.000031	0.0002105 ± 0.0000010
Measured	0.0001945 ± 0.0000012	0.031420 ± 0.000016	0.0002082 ± 0.0000021

Besides the confirmed accuracy of the measured U isotope ratios, also the achieved corresponding precision was appropriate for the aim of this study. While the expanded uncertainty (*k* = 2) for the *n*(^235^U)/*n*(^238^U) amount ratio was as low as 0.05%, this value increased to 0.62% and 1.01% for the minor U isotope ratios (Table 1).

The U isotope ratios of all samples reported here were additionally confirmed by independent in-house TIMS measurements. Taken together, all above experimental data proved beyond doubt the accuracy of the U isotope ratios measured with LA-MC-ICP-MS, applying the experimental protocol outlined above.

### 3.2 Spatial (in-)homogeneity of UO_2_ pellets from CMX-4

During CMX-4, organised by the International Technical Working Group on Nuclear Forensics (ITWG), two low-enriched UO_2_ pellets were delivered to each participating laboratory [3]. After initial non-destructive analysis using high resolution gamma spectrometry [9], fragments of ∼1.3 g of these two UO_2_ pellets were dissolved in 8 M HNO_3_ and subsequently analysed for their U isotopic composition using TIMS, following accredited analytical procedures [5]. These TIMS results served as a basis for the comparison to the LA-MC-ICP-MS measurements. It is important to note that both non-destructive high resolution gamma spectrometry and destructive TIMS analysis provided an average U isotopic composition of the bulk material only. Results of LA-MC-ICP-MS analysis in turn, had the potential to offer both spatially resolved as well as bulk U isotopic composition of the investigated UO_2_ pellets.

#### 3.2.1 Individual laser ablation spot analysis

Initial experiments employing LA-MC-ICP-MS included the U isotopic analysis of 10 individual spots of 5 μm diameter each of the two samples, denoted hereafter CMX-4-9-1 and CMX-4-9-3. After ablation of material from the surface, a certain amount of debris is formed around the actual crater at all times. Therefore, the laser beam was always moved at least 30 μm between individual spots to 1) avoid potential cross-contamination and 2) analyse an unaltered surface each time. The intention of this analytical approach was to investigate its applicability with respect to measurement precision of the individual U isotope ratios and subsequently to assess the intra-pellet homogeneity of the U isotopic composition. In other words this experiment aimed at revealing if the achievable measurement precision is sufficiently low to clearly indicate changes of the U isotopic composition between individual sample spots for each of the two UO_2_ pellets.

**Fig. 1:**
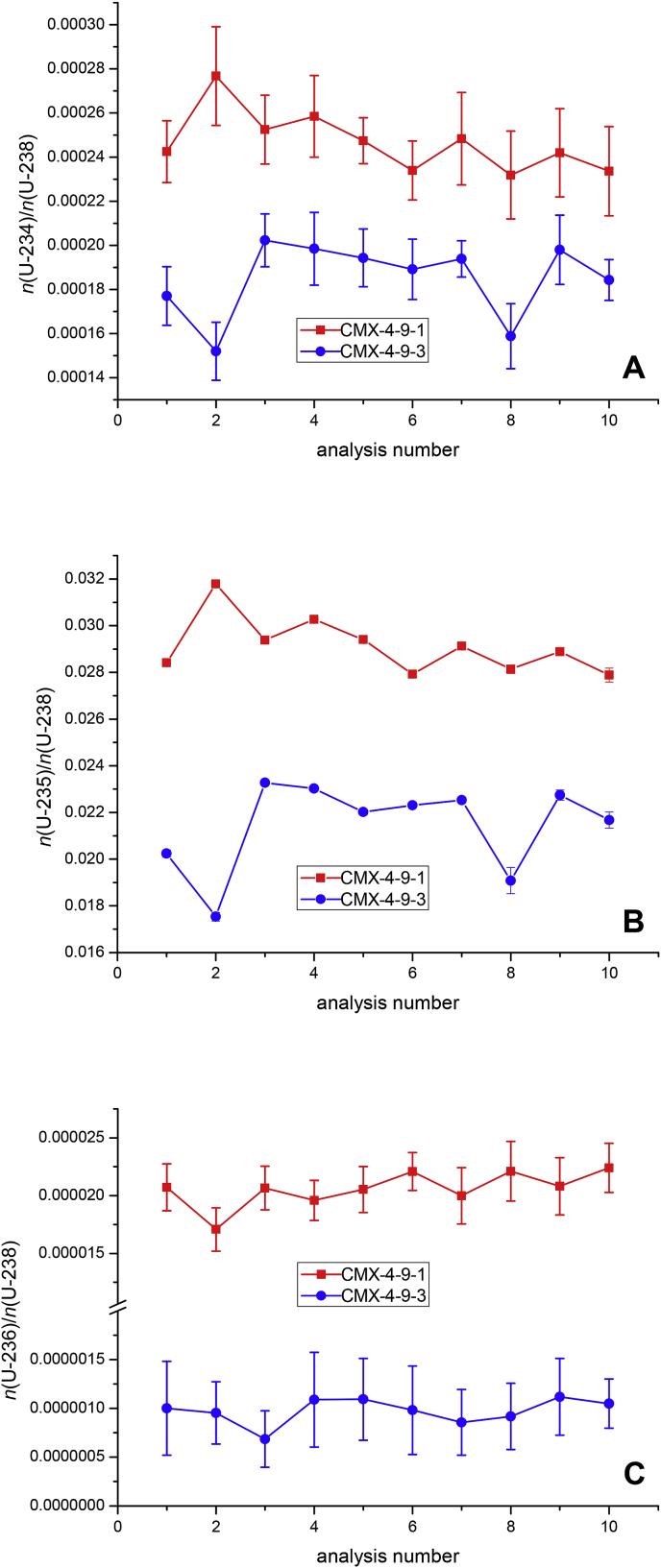
Sequence of 10 consecutive individual laser ablation spot analyses of two low-enriched UO_2_ pellets (∼2.2 wt% and ∼2.9 wt%) from the 4th Collaborative Materials Exercise (CMX-4) organised by the Technical Working Group on Nuclear Forensics (ITWG) employing MC-ICP-MS. The major (panel B) and minor (panels A and C) U isotope ratios highlight the inter- and intra-sample variability of samples CMX-4-9-1 and CMX-4-9-3.

Fig. 1 summarises the variability of the major and minor U isotope ratios in samples CMX-4-9-1 and CMX-4-9-3 as established by multiple single spot LA-MC-ICP-MS analysis. This data highlights the overall enrichment in ^235^U, being ∼2.9 wt% and ∼2.2 wt% for CMX-4-9-1 and CMX-4-9-3, respectively. On average the measurement precision of the *n*(^235^U)/*n*(^238^U) ratio of individual sample spots was well below 1%, while the between spot variability was much larger (Fig. 1 B). For the *n*(^234^U)/*n*(^238^U) ratio the average measurement precision of individual sample spots amounted to ∼7%, a value similar to the between spot variability for each of the two UO_2_ pellets (Fig. 1 A).

Because of the low abundance of ^236^U in the two UO_2_ pellets, the achievable measurement precision for the *n*(^236^U)/*n*(^238^U) ratio further deteriorated. The *n*(^236^U)/*n*(^238^U) ratio in CMX-4-9-1 was one order of magnitude smaller than the corresponding *n*(^234^U)/*n*(^238^U) ratio (Fig. 1C). Thus the average precision of individual spot analysis of the *n*(^236^U)/*n*(^238^U) ratio in sample CMX-4-9-1 increased to ∼10%. The *n*(^236^U)/*n*(^238^U) ratio in pellet CMX-4-9-3 was even one order of magnitude smaller, leading to an average measurement precision of only ∼39%. The spot-to-spot variability of the *n*(^236^U)/*n*(^238^U) ratio in both CMX-4-9-1 and CMX-4-9-3 was <10%, thus statistically not significant compared to the achieved measurement precision. In consequence, the applied LA-MC-ICP-MS measurement protocol proved inappropriate to assess spatial inhomogeneity of such low *n*(^236^U)/*n*(^238^U) ratios.

#### 3.2.2 Line scan analysis

**Fig. 2:**
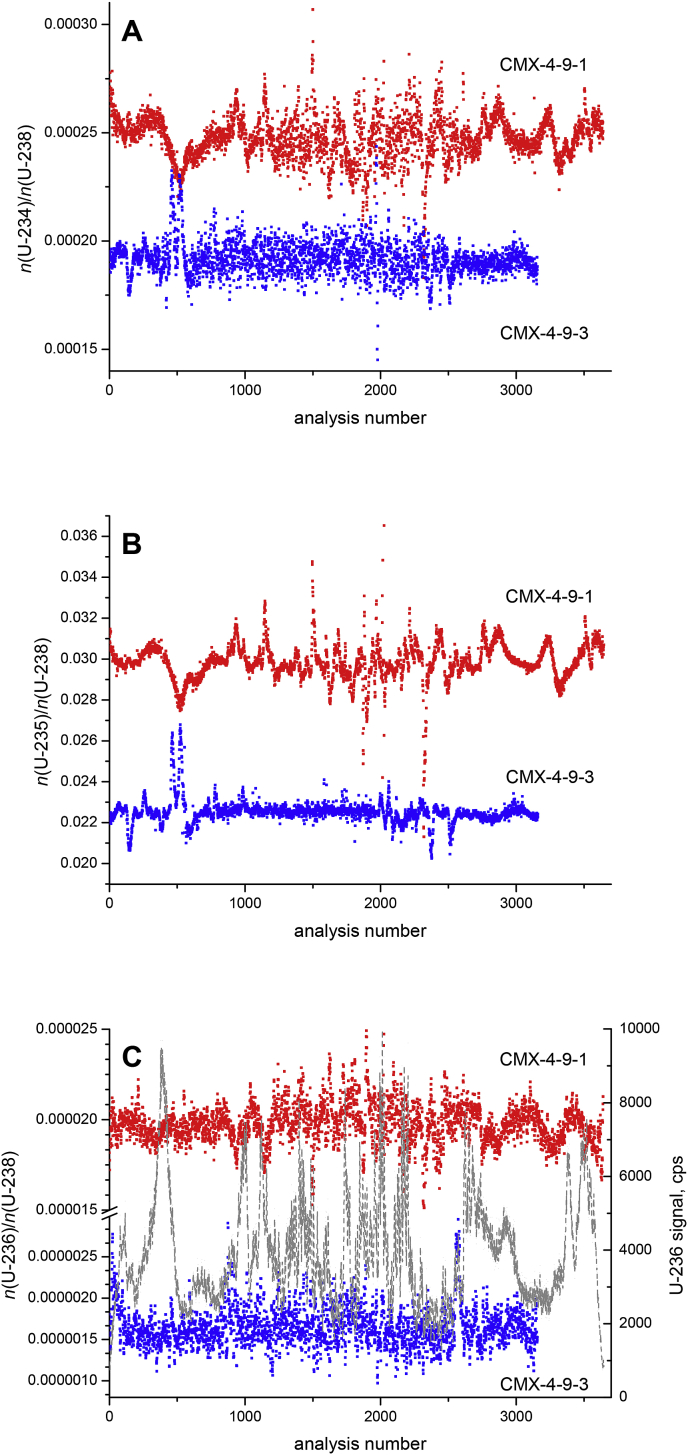
Line scan analysis of two low-enriched UO_2_ pellets revealing the spatial inhomogeneity with respect to their U isotopic composition. Samples CMX-4-9-1 and CMX-4-9-3 comprise 3618 and 3124 individual data points, respectively. A 5-point moving average was plotted for the minor U isotopes (panel A + C), to reduce the scatter of the individual data points. The dashed grey line in panel C represents the ion counter signal of ^236^U in counts per second (cps) during line scan analysis of sample CMX-4-9-1.

While LA analysis of individual sample spots already provided a good estimate of spatially resolved U isotopic information, line scan analysis allowed much deeper insights into the (in-)homogeneity of the U isotopic composition of the investigated UO_2_ pellets. For line scan analysis, spots of 5 μm diameter were ablated continuously for a couple of minutes across the surface of each of the two UO_2_ pellets yielding more than 3000 data points for pellets CMX-4-9-1 and CMX-4-9-3, respectively (Fig. 2). Focussing on the *n*(^235^U)/*n*(^238^U) ratio (Fig. 2B), it became evident that both examined UO_2_ pellets show distinct inhomogeneity. For CMX-4-9-1, the *n*(^235^U)/*n*(^238^U) ratio varied between 2.13 × 10^−2^ and 3.65 × 10^−2^, whereas these values only fluctuated from 2.02 × 10^−2^ to 2.68 × 10^−2^ for CMX-4-9-3. While both UO_2_ pellets were low-enriched in ^235^U, the isotopic inhomogeneity was more pronounced for CMX-4-9-1 (Fig. 2B). It is worth mentioning, however, that this observed within pellet variability of the *n*(^235^U)/*n*(^238^U) ratio of the two investigated UO_2_ pellets can be considered as relatively low and was much higher for other UO_2_ pellets investigated in an earlier study [21]. A quantitative assessment of this isotopic inhomogeneity, based on descriptive statistics and experimental data of additional samples, including two homogeneous certified U isotopic reference materials, is provided in section 3.4. Interestingly, most changes of the *n*(^235^U)/*n*(^238^U) ratio of CMX-4-9-1 occurred over longer distances (up to ∼1.8 mm), while these changes were generally much sharper for CMX-4-9-3 (up to 0.25 mm). This implies that pellet CMX-4-9-1 consists of regions of isotopic inhomogeneity that are on average distinctly larger than those of pellet CMX-4-9-3. Because CMX-4-9-1 shows not only broad but also sharp changes of the *n*(^235^U)/*n*(^238^U) ratio, this feature highlights (at least) two different components having different U enrichments, that have been mixed to produce this material (Fig. 2B).

Even though line scan analysis also satisfactorily disclosed the minor U isotope ratios, the limited precision of these measurements could only reveal small further details on potential U isotopic inhomogeneity (Fig. 2A + C). A part of the observed spread of the data was caused by the low ^234^U and ^236^U signals generated during the line scan measurements. Plots of individual minor U isotope ratios displayed largely scattering data. Therefore, a 5-point moving average was used in Fig. 2A + C. As such, additional information on the spatial distribution of the minor U isotopes is obtained.

Additionally, counting statistics influenced the achievable precision as is exemplified for line scan analysis of the *n*(^236^U)/*n*(^238^U) ratio of CMX-4-9-1 and CMX-4-9-3 (Fig. 2C): The dashed grey line in this figure displays the ^236^U signal intensity of pellet CMX-4-9-1 with the corresponding scale shown on the right y-axis. Ion counter signals for ^236^U varied between ∼1000 cps and ∼10 000 cps (Fig. 2C). It is evident that the measurement precision of the *n*(^236^U)/*n*(^238^U) ratio deteriorates when the ^236^U signal accounts for a few thousand cps only. Therefore any variation of the *n*(^236^U)/*n*(^238^U) ratio must be interpreted carefully because such a change might be just triggered by poor counting statistics (see below).

The signal intensities of ^236^U in CMX-4-9-3 were one order of magnitude lower than in CMX-4-9-1, varying between 65 cps and 1550 cps. The graphs displayed in Fig. 2C indicate that variation of the *n*(^236^U)/*n*(^238^U) ratio of CMX-4-9-3 was much smaller compared to that of CMX-4-9-1. Actually, essentially governed by counting statistics, the relative standard deviation of the average *n*(^236^U)/*n*(^238^U) ratio amounted to 11.2% for CMX-4-9-1, while this value deteriorated to 28.3% for CMX-4-9-3. Considering the lower limits of the range of the ^236^U signals of CMX-4-9-1 (1000 cps) and CMX-4-9-3 (100 cps), negligible uncertainty contributions of ^238^U measurements, as well as an acquisition time of 0.2 s per data point, pure counting statistics dictate a relative standard deviation on the *n*(^236^U)/*n*(^238^U) ratio of 7.1% and 22.4%. The difference between the RSD of the measured *n*(^236^U)/*n*(^238^U) ratio and the RSD resulting from counting statistics can be attributed to the isotopic inhomogeneity of the two UO_2_ pellets.

**Fig. 3:**
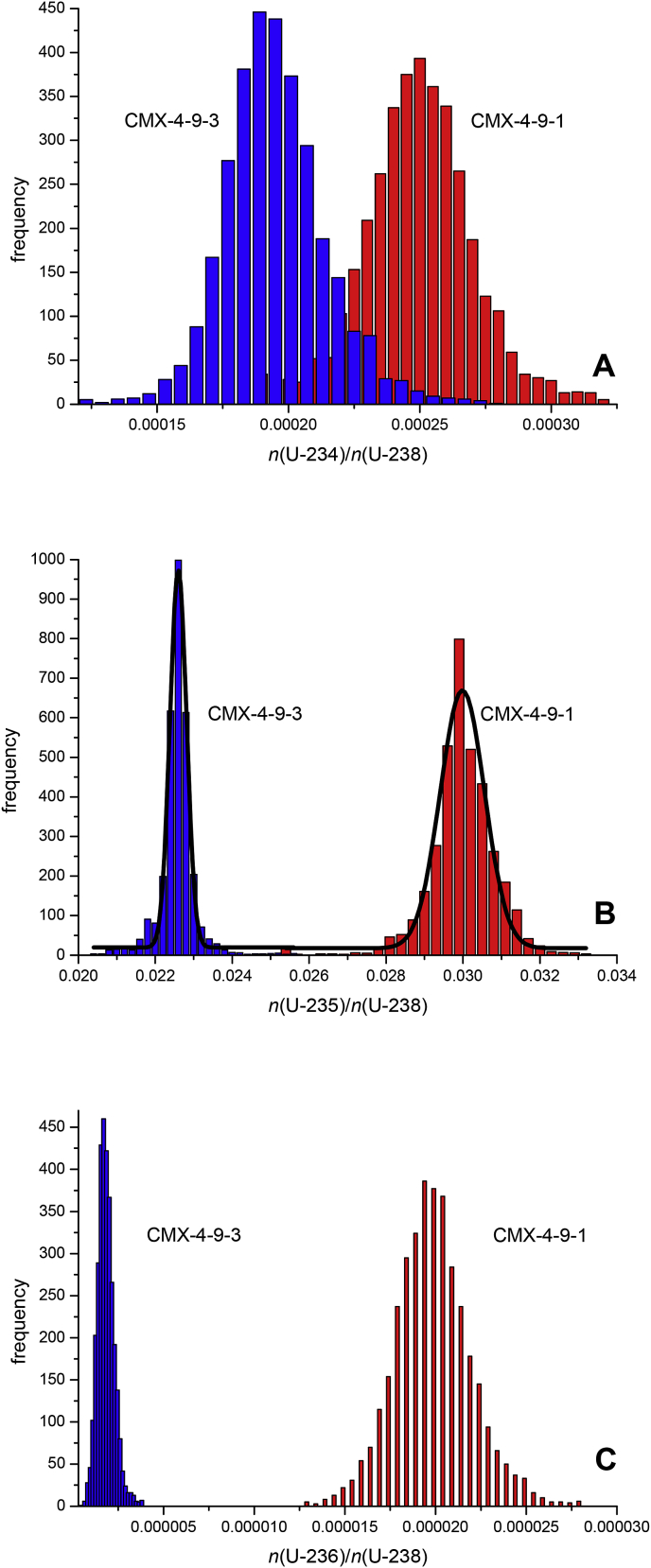
Frequency distribution of major and minor U isotope ratios in samples CMX-4-9-1 and CMX-4-9-3 highlighting the different U isotopic composition of both UO_2_ pellets. The bold solid lines in panel B highlight the normal fit of the respective frequency distributions of the *n*(^235^U)/*n*(^238^U) amount ratios. The larger half width of the normal fit of sample CMX-4-9-1 indicates larger isotopic inhomogeneity compared to UO_2_ pellet CMX-4-9-3. See text for details.

Because line scan analysis generated thousands of individual U isotope ratios of the investigated samples, representative frequency histograms of these results could be plotted straightforwardly. Fig. 3 summarises this data for the two UO_2_ pellets CMX-4-9-1 and CMX-4-9-3 visualising the differences in the distribution of the U isotopic composition of these two UO_2_ pellets. The added value of using frequency distributions of U isotope ratios over line scan analysis (Fig. 2) is the fact that the shape and width of the frequency distribution sheds additional light on the homogeneity and the possible production route of the investigated sample [21]. A narrow mono-modal frequency distribution of a specific U isotope ratio indicates an isotopically homogeneous material. Such distributions are represented by symmetric, Gaussian shape-like graphs [21]. Wide and asymmetric or bi-modal frequency distributions, in turn, indicate the presence of at least two isotopically different components that were mixed together. With respect to both UO_2_ pellets from CMX-4, the frequency distributions of all U isotope ratios were reasonably symmetric, with a minor tailing on the higher *n*(^235^U)/*n*(^238^U) ratio for both samples. This detail, together with the relatively broad frequency distribution of the *n*(^235^U)/*n*(^238^U) ratio indicated mixing of at least two starting materials of relatively close isotopic composition during the preparation of each of the UO_2_ pellets (e.g. in a powder blending process).

#### 3.2.3 Comparison of bulk and spatially resolved U isotopic analysis

Table 2 summarises the U isotopic composition of both UO_2_ pellets from CMX-4. This data was obtained via TIMS analysis as well as by LA-MC-ICP-MS analysis employing both line scan and individual spot analysis. Results are reported as mass fraction (wt%) with the associated expanded uncertainty with a coverage factor of 2. Overall, the three applied analytical approaches yielded very similar results. Nevertheless, the corresponding uncertainty of LA-MC-ICP-MS analysis appears considerably higher compared to those of the TIMS measurements (Table 2). This observation, however, is only true at first sight and can be explained as follows:

**Table 2:** Uranium isotopic composition (mass fraction ± uncertainty, wt%, *k* = 2) in two low-enriched UO_2_ pellets from the CMX-4 exercise using LA-MC-ICP-MS and comparison to bulk isotopic analysis using TIMS.

	Laser ablation analysis		TIMS
	Line scan	Individual spot analysis	
**CMX-4-9-1**			
^234^U	0.0237 ± 0.0045	0.0236 ± 0.0019	0.02454 ± 0.00074
^235^U	2.86 ± 0.18	2.79 ± 0.16	2.8597 ± 0.0023
^236^U	0.00190 ± 0.00043	0.00199 ± 0.00021	0.00180 ± 0.00018
^238^U	97.11 ± 0.17	97.18 ± 0.15	97.1137 ± 0.0037
**CMX-4-9-3**			
^234^U	0.0188 ± 0.0039	0.0178 ± 0.0024	0.01852 ± 0.00056
^235^U	2.20 ± 0.11	2.07 ± 0.26	2.1698 ± 0.0018
^236^U	0.00016 ± 0.00009	0.00009 ± 0.00002	<0.001
^238^U	97.80 ± 0.11	97.91 ± 0.26	97.8110 ± 0.0037

Individual spot analysis consisted of 10 independent LA analyses of 5 μm spots on the surface of each pellet. Line scan analysis included 3618 and 3124 data points for CMX-4-9-1 and CMX-4-9-3, respectively, obtained while moving the laser beam with a speed of 20 μm s^−1^ across the sample surface for several minutes.

For TIMS analysis of a UO_2_ pellet, a sample mass of >500 mg is normally dissolved and chemically prepared according to our laboratory's accredited procedure. The uranium is then subjected to high precision TIMS measurement with filament loadings of 80 ng in total evaporation mode. The LA-MC-ICP-MS set-up employed here typically requires only 5 ng of material for a single measurement [20]. This is not only 5 orders of magnitude less material that is needed for LA-MC-ICP-MS, but also emphasises the boundary conditions of the two analytical approaches. The relatively large amount of sample employed for TIMS analysis will ultimately lead to an averaged U isotopic composition representing the overall bulk sample, measured with high accuracy and precision.

LA-MC-ICP-MS, in turn, sub-samples only minute amounts of the entire sample. If an investigated specimen is not homogeneous with respect to its U isotopic composition on a μm-scale, then single spot LA-MC-ICP-MS analysis will not provide results representative of the bulk material. Increasing the spot diameter, and averaging the results of the analysis of several individual LA spots will help this situation. However, depending on which spots are selected randomly for analysis, obtained results will vary profoundly. Line scan analysis, employing thousands of data points, will improve this situation fundamentally. Because of the far larger sample surface area considered, the obtained results of line scan analysis are much closer to the average value of TIMS analysis representing the bulk material.

While the following essentially holds true for all U isotope abundances, the determination of the mass fraction of ^235^U in sample CMX-4-9-1 may serve as an example to illustrate the above mentioned facts (Table 2). The overall abundance of ^235^U in this UO_2_ pellet determined via TIMS amounted to 2.8597 wt% ± 0.0023 wt%. Analysis of ten randomly-selected individual LA spots gave a value of 2.79 wt% ± 0.16 wt% of ^235^U. It is important to note, however, that the standard deviation of the individual results was far smaller (∼0.5% relative standard deviation, RSD) than the standard deviation (5.7% RSD, spot to spot variability) of the average ^235^U abundance between individual LA spots (Fig. 1B). As these 10 sampling points were not necessarily representative for the average U isotopic composition of the bulk material, the average ^235^U abundance is statistically not significantly different from the corresponding TIMS value.

The 3618 data points acquired during line scan analysis gave 2.86 wt% ± 0.18 wt% of ^235^U. This average ^235^U abundance was very close to that obtained using TIMS. The large number of individual data points generated during line scan analysis was increasingly representing the U isotopic composition of the bulk material. The uncertainty associated with the average ^235^U abundance of line scan analysis actually reflected the spatial ^235^U inhomogeneity within the sample. This high uncertainty was not caused by poor analytical measurements, but reflects the changes in the ^235^U abundance while scanning with the LA along the sample surface. It is worth to stress that for homogeneous material prepared from certified reference material much lower standard deviation was observed (<1% relative) Table 1) [21].

In summary, repetitive individual spot analysis provided straightforwardly a good estimate of the within pellet (in-)homogeneity of a UO_2_ pellet with respect to its *n*(^235^U)/*n*(^238^U) ratio. The average *n*(^235^U)/*n*(^238^U) ratio obtained this way, however, is not necessarily representative for the entire sample (Table 2). Also the *n*(^234^U)/*n*(^238^U) ratio revealed significant variability within individual UO_2_ pellets. The very low abundance of ^236^U in UO_2_ pellets manufactured from non-recycled uranium and in consequence too poor measurement precision, did not allow the detection of the spatial inhomogeneity of the *n*(^236^U)/*n*(^238^U) ratio itself. Average ^236^U abundance, however, could be established in these samples. Either ablating larger spots (larger sample surface area) or increasing the power of the laser (larger sample volume) or even both options would largely improve this constraint; however, at the expense of reduced spatial resolution.

Another practical limitation arising from either option is the fact that increasing ion counter signals for ^234^U and ^236^U would lead to a saturation of the Faraday detector measuring the ^238^U ion current. Having established the *n*(^235^U)/*n*(^238^U) in a separate measurement, the signals of both ^234^U and ^236^U could be measured with higher laser power and subsequently normalised to ^235^U. As such, the minor U isotopes ratios can be established with increased precision without saturating the Faraday detector measuring ^238^U. However, if all U isotopes (^234^U, ^235^U, ^236^U and ^238^U) are to be measured simultaneously such as in this study, then the maximum workable signal originating from the abundance of ^238^U in a specific sample is the factor governing the precision achievable for the signals of the minor U isotopes.

TIMS analysis yielded values of the U isotopic composition of the bulk material with small uncertainties. Line scan analysis using LA-MC-ICP-MS also gave accurate average U isotopic abundances, however, with a larger scatter in the data. The latter reflected the actual spatial inhomogeneity of the U isotopic composition of the investigated samples and was not a result of poor measurement precision.

#### 3.2.4 Comparison to previous U isotopic analysis of CMX-4 UO_2_ pellets

As the two low-enriched UO_2_ pellets CMX-4-9-1 and CMX-4-9-3 were part of the fourth Collaborative Materials Exercise (CMX-4), their U isotopic composition was also measured by the participating laboratories using either TIMS or ICP-MS [5] or gamma spectrometry [9]. These results, summarised recently [5,9], essentially confirm the U isotopic abundances reported here and as such present another independent check of the accuracy of the LA-MC-ICP-MS. Participating laboratories using TIMS or ICP-MS dissolved the UO_2_ pellets, prior to any mass spectrometric measurement. For gamma spectrometry the entire pellet was employed [9]. In this respect LA-MC-ICP-MS data presented here nicely complemented the bulk analysis methods, providing timely information on the spatial distribution of the U isotopic composition within each of the two UO_2_ pellets.

An indication of potential heterogeneity of the U isotopic composition of the CMX-4 UO_2_ pellets was gained through secondary ion mass spectrometry (SIMS) analysis collecting particle from the pellets by swiping the surfaces [22]. Implementing an automated particle measurement (APM) software, preliminary SIMS analysis confirmed the frequency distribution of the ^235^U enrichments of the two CMX-4 UO_2_ pellets established with LA-MC-ICP-MS in this study (Fig. 3). Subsequent SIMS micro beam measurements on up to 40 micro particles yielded additional in-depth insights into the U isotopic composition of the two UO_2_ pellets. Plotting ^234^U/^238^U *vs.* ^235^U/^238^U atom ratios revealed that the results for all ∼40 analysed micro particles follow the same correlation line, suggesting that both pellets were prepared from uranium originating from employing the same enrichment process [22].

### 3.3 LA-MC-ICP-MS U isotopic analysis of selected U materials

Both individual spot analysis and line scan analysis were employed to characterise the U isotopic composition of four low-enriched UO_2_ pellets of different origin (hereafter referred to “pellet 1″ to “pellet 4″) as well as one seized depleted U metal sample (Table 3). The ^235^U enrichment of the four UO_2_ pellets varied from ∼1.8 wt% to ∼3.6 wt%. The U metal was depleted to ∼0.38 wt% with respect to ^235^U. All five investigated samples contained measurable amounts of ^236^U indicating the use of or cross-contamination with recycled (reprocessed) U during the preparation of the seized materials. Generally LA-MC-ICP-MS and TIMS results agreed well with each other and confirmed the high accuracy of the entire analytical data set.

**Table 3:** Comparison of U isotopic composition (mass fraction ± uncertainty, wt%, *k* = 2) of four low-enriched UO_2_ pellets and one seized depleted U metal sample determined using LA-MC-ICP-MS as well as TIMS bulk isotopic analysis.

	Line scan	Individual spot analysis	TIMS
**UO**_**2**_**pellet 1**			
^234^U	0.0147 ± 0.0028	0.0150 ± 0.0002	0.0150 ± 0.0004
^235^U	2.006 ± 0.024	2.0051 ± 0.0056	2.0048 ± 0.0010
^236^U	0.0083 ± 0.0018	0.0066 ± 0.0007	0.0069 ± 0.0007
^238^U	97.971 ± 0.023	97.9733 ± 0.0055	97.9726 ± 0.0028
**UO**_**2**_**pellet 2**			
^234^U	0.0274 ± 0.0070	0.02846 ± 0.00096	0.027772 ± 0.000026
^235^U	3.602 ± 0.017	3.594 ± 0.010	3.6016 ± 0.0026
^236^U	0.040 ± 0.010	0.0426 ± 0.0015	0.03965 ± 0.00016
^238^U	96.331 ± 0.016	96.3349 ± 0.0099	96.3310 ± 0.0023
**UO**_**2**_**pellet 3**			
^234^U	0.0148 ± 0.0025	0.01521 ± 0.00043	0.014917 ± 0.000024
^235^U	2.0053 ± 0.0086	2.0043 ± 0.0039	2.0056 ± 0.0014
^236^U	0.0066 ± 0.0015	0.00666 ± 0.00094	0.006512 ± 0.000029
^238^U	97.9732 ± 0.0085	97.974 ± 0.0039	97.9730 ± 0.0013
**UO**_**2**_**pellet 4**			
^234^U	0.0143 ± 0.0005	0.0140 ± 0.0005	0.0143 ± 0.0015
^235^U	1.802 ± 0.016	1.8030 ± 0.0024	1.8017 ± 0.0091
^236^U	0.0196 ± 0.0065	0.0192 ± 0.0007	0.0197 ± 0.0020
^238^U	98.165 ± 0.016	98.164 ± 0.002	98.164 ± 0.042
**U metal**			
^234^U	0.00234 ± 0.00096	0.00238 ± 0.00018	0.002153 ± 0.000005
^235^U	0.384 ± 0.029	0.3872 ± 0.0051	0.3801 ± 0.0003
^236^U	0.0028 ± 0.0011	0.00290 ± 0.00028	0.00267 ± 0.00001
^238^U	99.611 ± 0.029	99.608 ± 0.005	99.615 ± 0.042

Individual spot analysis consisted of 10 independent LA analyses of 5 μm spot on the surface of each sample. Line scan analysis included several thousand data points for each sample, obtained while moving the laser beam with a speed of 20 μm s^1^ across the surface for several minutes.

Among the applied analytical approaches, results of line scan analysis had the highest uncertainty, followed by individual spot analysis and TIMS (Table 3). As mentioned earlier, line scan analysis records fluctuations of the U isotopic composition of a sample while moving the laser beam across its surface. This variation of the U isotopic composition may serve as an indicator of the isotopic homogeneity of such specimens. Comparing the uncertainty of the mass fractions of ^235^U and ^238^U of the five samples in Table 3 to the corresponding data of the two UO_2_ pellets from CMX-4 (Table 2), it is evident that the former is about one order of magnitude lower. This indicates that the four UO_2_ pellets and the depleted U metal are isotopically distinctly more homogeneous than the two UO_2_ pellets from CMX-4. However, this feature requires a quantitative measure to express its inhomogeneity using statistical assessments.

### 3.4 Quantitative assessment of isotopic homogeneity of investigated uranium samples

As shown above, the *n*(^235^U)/*n*(^238^U) amount ratio obtained via line scan analysis is the most powerful indicator of U isotopic (in-)homogeneity of a sample. Employing this U isotope ratio and calculating descriptive statistical parameters such as skewness and half width (expressed as FWHM) from the fitted data, a quantitative statement of the degree of spatial inhomogeneity was aimed at.

The statistical parameter skewness quantifies the extent to which a given distribution differs from a normal distribution. The latter can be reasonably assumed for the frequency distribution of the *n*(^235^U)/*n*(^238^U) amount ratio in a homogeneous material. This is reflected by a skewness between −0.5 and 0.5 [25]. If skewness is between −1 and −0.5 or between 0.5 and 1, the distribution is moderately skewed. All skewness values beyond −1 or 1 describe highly skewed data. The skewness calculated from the *n*(^235^U)/*n*(^238^U) ratios obtained from line scan analysis of the U samples investigated in this study is summarised in Table 4.

**Table 4:** Descriptive statistical parameters (skewness, half width) of the *n*(^235^U)/*n*(^238^U) ratio of eight low-enriched UO_2_ pellets, one depleted U metal, and two certified isotopic reference materials as determined using line scan analysis.

Sample name	Skewness	Half width^a^
CMX-4-9-1	−1.1319	0.00136
CMX-4-9-3	2.4226	0.000508
UO_2_ pellet 1	0.8513	0.000126
UO_2_ pellet 2	−0.0269	0.000167
UO_2_ pellet 3	0.1675	0.000088
UO_2_ pellet 4	1.1920	0.000147
U metal	0.0526	0.000143
		
CMX-5/1^b^	2.7731	0.000817
CMX-5/2^b^	1.4554	0.00404

*Certified reference materials*^b^		
CRM-125A	0.1716	0.000180
U-010	0.3506	0.0000828

a The experimental data were fitted with a Gaussian function from which the half width was calculated as full width at half maximum (FWHM).

b CMX-5/1 and CMX-5/2 are two low-enriched UO_2_ pellets from the ITWG's 5^th^ Collaborative Materials Exercise. The two certified reference materials CRM-125A (sintered UO_2_ pellet) and U-010 (pressed pellet from powder) serve as a reference point for the assessment of the isotopic homogeneity of the investigated samples. These four samples were analysed previously [21]. This experimental data is also used here to have a more solid dataset for comparison.

Additionally, the skewness was also calculated from experimental data of an earlier investigation [21], namely two UO_2_ pellets and two certified U isotopic reference materials (Table 4). As the two employed certified U isotopic reference materials CRM-125A and U-010 are known to be homogenous with respect to their U isotopic composition, the skewness of their distribution of the analysed *n*(^235^U)/*n*(^238^U) amount ratio may serve as a solid basis for the assessment of the homogeneity of unknown samples. In fact, the skewness of both certified reference materials is well between −0.5 and 0.5, which is the statistical criterion for a distribution that is close to a normal distribution. The U metal and two of the UO_2_ pellets (pellet 2 and 3) also fulfil this criterion, while the remaining samples (pellet 1 and 4) have a higher skewness. As the ^235^U and ^238^U mass fractions of the UO_2_ pellets 1–4 and the U metal have very similar uncertainties, the skewness alone does not reflect sufficiently the homogeneity of these samples.

Therefore, the half width (defined as full width at half maximum, FWHM) of the frequency distribution of the *n*(^235^U)/*n*(^238^U) amount ratio was considered as additional statistical parameter. To this end, a one-component normal distribution was fitted using the experimental data of line scan analysis. This is a simplification because the skewness determined above revealed that some of the data sets do not strictly follow a Gaussian distribution. However, this approached is considered fit-for-purpose and consequently allows the calculation of the half width of the fitted normal distribution.

The half widths of the normal distribution of the *n*(^235^U)/*n*(^238^U) amount ratio of the two well-characterised reference materials were calculated. These half widths were very small and varied between ∼8 × 10^−5^ and ∼2 × 10^−4^ (Table 4). The smaller half width of U-010 indicates higher homogeneity of this CRM, prepared by UF_6_ hydrolysation, compared to CRM 125-A that was produced via powder blending. This narrow span of half widths may serve as useful comparison for the evaluation of the other samples of this dataset. In fact, the half width of both the four UO_2_ pellets 1–4 and the depleted metal are also within this narrow range. Therefore, these samples can be considered homogeneous with respect to their U isotopic composition.

In contrast, the half widths of the normal fit of the frequency distribution of the four UO_2_ pellets of CMX-4 and CMX-5 were clearly exceeding those of the two certified reference materials. The half width also provided a good indication for distinguishing the degree of inhomogeneity between these samples: the normal distribution of the frequency distribution of the *n*(^235^U)/*n*(^238^U) amount ratio of UO_2_ pellet CMX-4-9-3 had ∼50% of the half width of that of CMX-4-9-1, indicating that the latter is isotopically less homogeneous (Fig. 3B). This finding is also illustrated in the line scan analysis displayed in Fig. 2B. Similar dissimilarities of the half widths are observed for the two CMX-5 UO_2_ pellets (Table 4 [21]).

Both skewness and half width provide a numerical description of the isotopic composition. While both parameters are low for the two certified reference materials, skewness and half width provide a consistent picture of the homogeneity of pellet 1 and pellet 4. The datasets of the most inhomogeneous samples (CMX-4 and CMX-5) shared the highest skewness. Overall, the half width allowed a better distinction between the different levels of isotopic inhomogeneity of various samples. While the distances of isotopic inhomogeneity differed almost 10-fold between the two CMX-4 samples (up to ∼1.8 mm for CMX-4-9-1 and up to 0.25 mm for CMX-4-9-3) (Fig. 2B), the difference in length of isotopic inhomogeneity of the two UO_2_ pellets from CMX-5 was less pronounced. The distance between various areas of U isotopic homogeneity varied between ∼0.5 mm and ∼0.8 mm for CMX-5/1 and CMX-5/2, respectively.

## 4 Conclusions

The accurate U isotopic composition of solid U bearing materials was established through repeated LA of the sample surface and subsequent analysis via MC-ICP-MS. The employed LA cell helped produce highly spatially-resolved U isotope data. Among all measured U isotope ratios, the *n*(^235^U)/*n*(^238^U) ratio provided the most useful signature to examine the spatial (in-)homogeneity of U samples on a μm-scale. Descriptive statistical parameters such as skewness and half width allowed a quantitative assessment of the corresponding isotopic inhomogeneity.

Taken together, the developed LA-MC-ICP-MS protocol proved to be a powerful, straightforward tool to assess U isotopic inhomogeneity quantitatively in uranium bearing materials with high accuracy. This can allow drawing conclusions on the process used for pellet production (e.g. powder blending or precipitation) and on the starting materials (i.e. initial isotopic composition of the components of the blend). While bulk analytical techniques such as solution-based MC-ICP-MS or TIMS offer superior uncertainty, micro-analytical techniques such as LA-MC-ICP-MS provide data from which indications on the production processes can be inferred. This valuable information essentially supports nuclear forensic investigations.

## Declaration of interests

The authors declare that they have no known competing financial interests or personal relationships that could have appeared to influence the work reported in this paper.
